# Balancing risks of surgical complications and positive margins for patients with invasive lobular carcinoma of the breast and elevated BMI: An institutional cohort study

**DOI:** 10.1016/j.amjsurg.2024.116073

**Published:** 2024-11-06

**Authors:** Israel Falade, Kayla Switalla, Astrid Quirarte, Molly Baxter, Daniel Soroudi, Harriet Rothschild, Shoko Emily Abe, Karen Goodwin, Merisa Piper, Michael Alvarado, Bao-Quynh Julian, Cheryl Ewing, Jasmine Wong, John Rose, Laura Esserman, Robert Foster, Rita A. Mukhtar

**Affiliations:** a University of California San Francisco, School of Medicine, San Francisco, CA, USA; b University of California San Francisco, Department of Surgery, San Francisco, CA, USA

## Abstract

**Background:**

The risks of postoperative complications in breast cancer patients vary by patient and tumor characteristics. Elevated BMI and invasive lobular carcinoma (ILC) increase risks of surgical complications and positive margins, respectively.

**Methods:**

We retrospectively analyzed patients with BMI ≥30 kg/m^2^ from an institutional ILC database. The primary outcome was surgical complication rate by procedure type. The secondary outcome was positive margin rates by surgical approach, stratified by T stage.

**Results:**

Of 154 analyzed patients, standard BCS, lumpectomy with oncoplastic closure, and simple mastectomy had the lowest complication rates (18.2 %, 17.0 %, 11.8 %). Oncoplastic reduction mammoplasty and mastectomy with aesthetic closure had the highest rates (35.5 %, 33.3 %). The overall positive margin rate was 28.5 %, significantly higher in BCS vs. mastectomy (37.4 % vs. 15.0 %, p = 0.003). Oncoplastic surgery significantly reduced positive margin rates in BCS.

**Conclusion:**

In this study, 23.4 % of patients experienced surgical complications, with higher rates in oncoplastic/reconstructive approaches. However, oncoplastic surgery reduced positive margins, highlighting the importance of balancing risks for optimal surgical planning.

## 1. Introduction

Patients diagnosed with breast cancer must make several decisions regarding treatment, including selecting the type of surgery to undergo. Surgical resection of the primary tumor remains an important component of breast cancer treatment, with the two main surgical options being breast conservation (also known as lumpectomy), or mastectomy.^1^ Within these broad categories exist additional variations: patients can undergo standard breast conserving surgery (BCS), or alternatively combine BCS with oncoplastic approaches to improve the aesthetic outcome. For those undergoing mastectomy, options include simple mastectomy without reconstruction, mastectomy with aesthetic flat closure, or mastectomy with reconstruction (which may be implant or tissue based).^2^(see Table 1 )

Two of the main early outcomes to consider when gauging the effectiveness of breast cancer surgery include positive margin rates, and surgical complication rates.^3^ While more extensive surgery such as mastectomy can reduce the risk of positive margins, it may result in increased risk of other surgical complications.^4^ The pros and cons of each surgical choice vary based on both patient factors and tumor specific factors.

For patients with elevated body mass index (BMI), which impacts approximately 42 % of the population, the risk of surgical complications across a wide array of procedures is elevated.^5^ Indeed, many reconstructive procedures are not offered above certain BMI thresholds due to data showing increased surgical complications.^6^ Data show that breast cancer patients with BMI above 30 kg/m^2^ are less likely to undergo reconstruction after mastectomy, and obesity has been viewed as a potential contraindication to breast reconstruction.^7,8^

Tumor type can also influence surgical outcomes. For example, patients with the second most common type of breast cancer, invasive lobular carcinoma (ILC), face elevated risk of positive margins at surgical resection.^9^ Comprising 10–15 % of all breast cancer diagnoses, ILC grows in a diffuse pattern due to its characteristic absence of the adhesion protein E-cadherin.^10^ Several studies report that the risk of positive margins following BCS can exceed 40 % in patients with ILC.^11–14^ We and others have shown that the addition of oncoplastic surgery to BCS may reduce the risk of positive margins in those with ILC, but the impact of adding such techniques on other surgical complications for these patients is unknown.^15,16^

For these reasons, surgical decision making for women with ILC and elevated BMI is especially challenging, with no published data to our knowledge reporting outcomes in this group specifically. We therefore evaluated an institutional cohort of patients with ILC and BMI ≥30 kg/m^2^ and determined the incidence of surgical complications and positive margin rates by type of breast surgery performed. The goal of this analysis is to provide data for surgeons and patients that may help inform surgical decision making when balancing the risks and benefits of various surgical approaches.

## 2. Methods

### 2.1. Study design and data collection

We retrospectively selected a cohort of consecutive patients with a BMI ≥30 kg/m^2^ from a prospectively maintained institutional database of patients undergoing surgical treatment for ILC between 1995 and 2023. The BMI threshold was selected based on the World Health Organization definition of obesity.^17^ Patients were excluded if they had *de novo* stage IV disease, lacked BMI data at the time of surgery, or did not undergo surgical treatment. This retrospective study was approved by the University of California, San Francisco Institutional Review Board (IRB).

Clinicopathological information including tumor size, tumor (T) category, BMI, type of surgical operation, use of oncoplastic surgery, use of shave margins, and rate of positive surgical margins were collected from the database. Patients were grouped by initial breast surgery procedure performed, as either BCS or mastectomy. Within the BCS group, patients were further categorized as undergoing standard BCS, or BCS with oncoplastic surgery (either oncoplastic closure or oncoplastic reduction mammoplasty [ORM]). Within the mastectomy group, patients were categorized as undergoing simple mastectomy (mastectomy without reconstruction), mastectomy with aesthetic flat closure, or mastectomy with reconstruction (skin sparing [SSM] or total/nipple sparing [TSSM]) with either implant based or autologous reconstruction. Mastectomy with aesthetic flat closure was defined as the removal of extra skin and fat after the mastectomy, followed by shaping and smoothing the remaining tissue to create a flat chest contour (performed by a plastic surgeon in this cohort). Procedure type was determined by review of operative reports. The use of shave margins was determined by review of pathology reports, with resection of any additional margin being considered a shave margin. Of note, routine cavity shave margin use has been recommended at our institution for patients with ILC undergoing BCS since 2018; use of shave margins at the time of mastectomy is performed selectively per preference of the treating surgeon.

The primary outcome was the rate of surgical complications for each procedure type overall and stratified by T category. Surgical complications included any of the following occurrences diagnosed within 6 months of primary breast surgery: infection, seroma, hematoma, poor wound healing, or unplanned return to the operating room. The presence of surgical complications was determined from review of the medical record and provider notes. Infection was defined by documentation in the medical record of “infection,” “abscess,” “breast cellulitis,” “infected implant,” or “donor site infection” and treatment with antibiotics.

Seroma and hematoma were defined as complications if a healthcare provider documented seroma or hematoma as a clinical diagnosis, irrespective of subsequent interventions for management. Poor wound healing included documentation of “skin flap necrosis,” “muscle flap necrosis,” “incisional breakdown,” “non-healing wound,” or “wound dehiscence” in post-operative hospital visit records. An unplanned return to the operating room was defined as any additional procedure required for a complication resulting directly or indirectly from the primary operation.

The secondary outcome was positive margin rate by type of surgical procedure. Margins were considered positive if pathologic evaluation of final margins indicated “ink on tumor.” Additional outcomes evaluated included total number of oncologic operations performed for primary tumor and their associated complication rates.

### 2.2. Statistical analysis

Patient and tumor characteristics were compared by procedure type using t-tests and Pearson’s chi-square tests. We used summary statistics to evaluate complication rates and positive margin rates. Chi-square tests were used to analyze the associations between surgical procedure type and complication rates, as well as positive margin rates. Averaged total number of operations and complications for each patient were compared across surgical procedures using ANOVA. Logistic regression models were developed to evaluate predictors of surgical complications and positive margins. All analyses were performed using STATA 18.0 (Stata Corp., College Station, TX, USA), with two-tailed p < 0.05 indicative of statistical significance.

## 3. Results

### 3.1. Overall demographics

Of 804 ILC patients in the institutional database, we identified a cohort of 154 patients with stage I-III ILC and BMI ≥30 kg/m^2^. Patients in this cohort underwent their initial surgical procedures between 1999 and 2023. Average age was 62.9 α 11.6 years and average BMI was 34.9 kg/m^2^ (range 30.0–61.4 kg/m^2^). Most patients (55.6 %) had stage 2 disease, 86.0 % had hormone receptor (HR) positive and human epidermal growth factor receptor-2 (HER2) negative tumors, and 68.9 % had grade 2 tumors.

Overall, 92 patients (59.7 %) underwent BCS and 62 (40.3 %) underwent mastectomy as their first oncologic operation. Specifically, 35.7 % (n = 55) underwent standard BCS, 24.0 % (n = 37) underwent BCS with oncoplastic surgery (6 with oncoplastic closure and 31 with ORM), 11.0 % (n = 17) underwent simple mastectomy, 5.8 % (n = 9) underwent mastectomy with aesthetic flat closure, and 23.4 % (n = 36) underwent mastectomy with reconstruction (19 with SSM and 17 with TSSM). Among those who underwent SSM or TSSM, 75.0 % (n = 27) had tissue expanders and 25 % (n = 9) had immediate autologous reconstruction. Patients who underwent BCS as their first operation had significantly smaller tumor size compared to those who underwent mastectomy (mean 3.0 cm versus 5.1 cm, p < 0.01), but did not otherwise significantly differ in age, BMI, or ILC tumor grade. Shave margin use was common in this study cohort, with shave margins utilized in 76.5 % of patients undergoing BCS and 43.5 % of patients undergoing mastectomy. The use of oncoplastic and reconstructive approaches increased over time (p = 0.004), but there was no difference in complication rates or positive margins rates by time period.

### 3.2. Complication rates by procedure type

In this cohort of 154 patients with ILC and elevated BMI, 20.8 % of patients had one complication after their first oncologic procedure, while 9.1 % had more than one complication. Of these, the most common complication was infection, occurring in 13.6 % of patients, followed by seroma (9.1 %), poor wound healing (5.8 %), unplanned return to the operating room (5.8 %), and hematoma (3.9 %). Of those with seromas, 20 % required aspiration while the remaining were managed expectantly; for those with hematomas, 66.7 % underwent intervention. The overall complication rates in the BCS and mastectomy groups were 23.9 % and 24.2 % respectively.

**Fig. 1.**
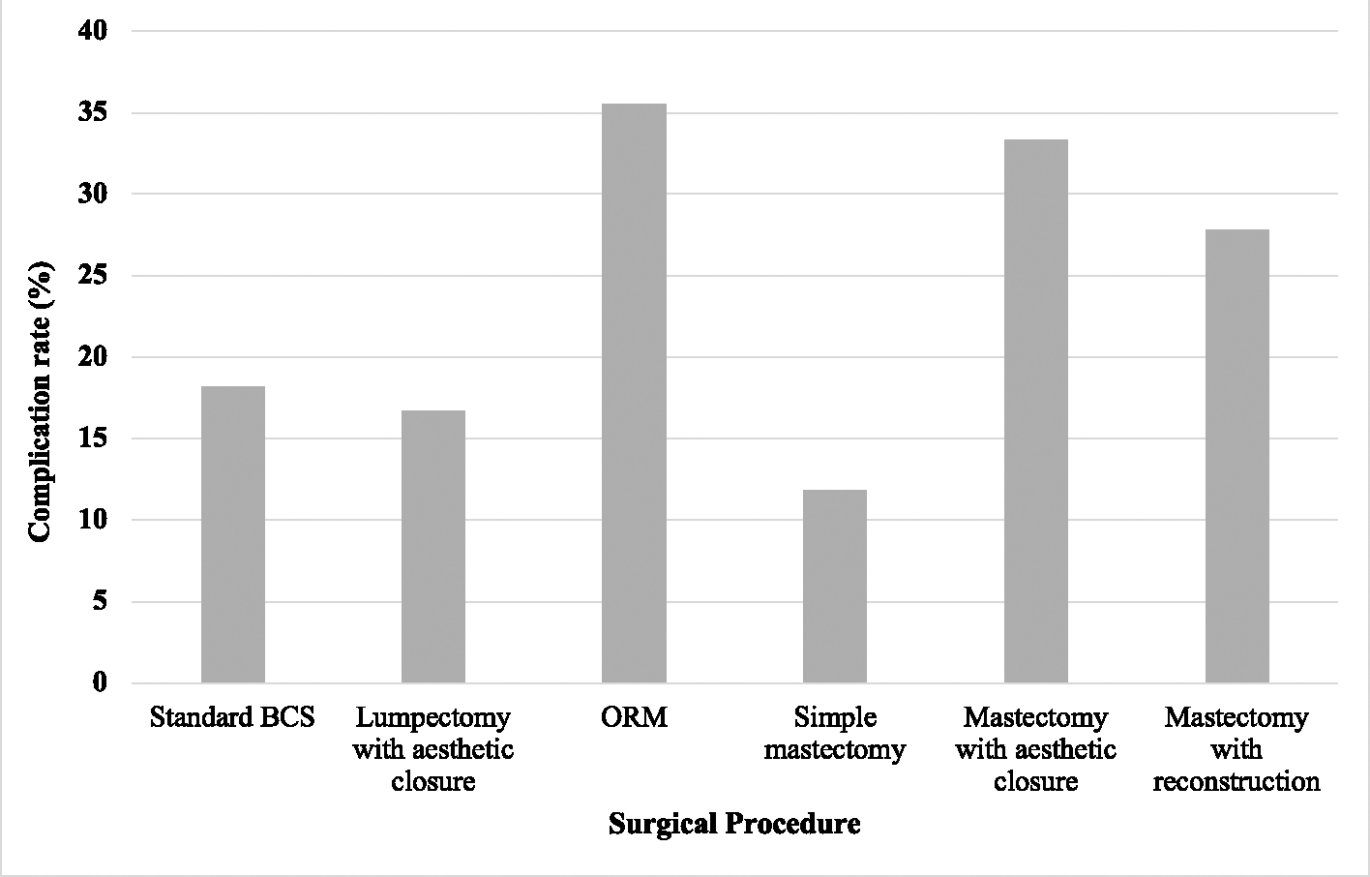
Complication rate across initial surgical procedure.

**Fig. 2.**
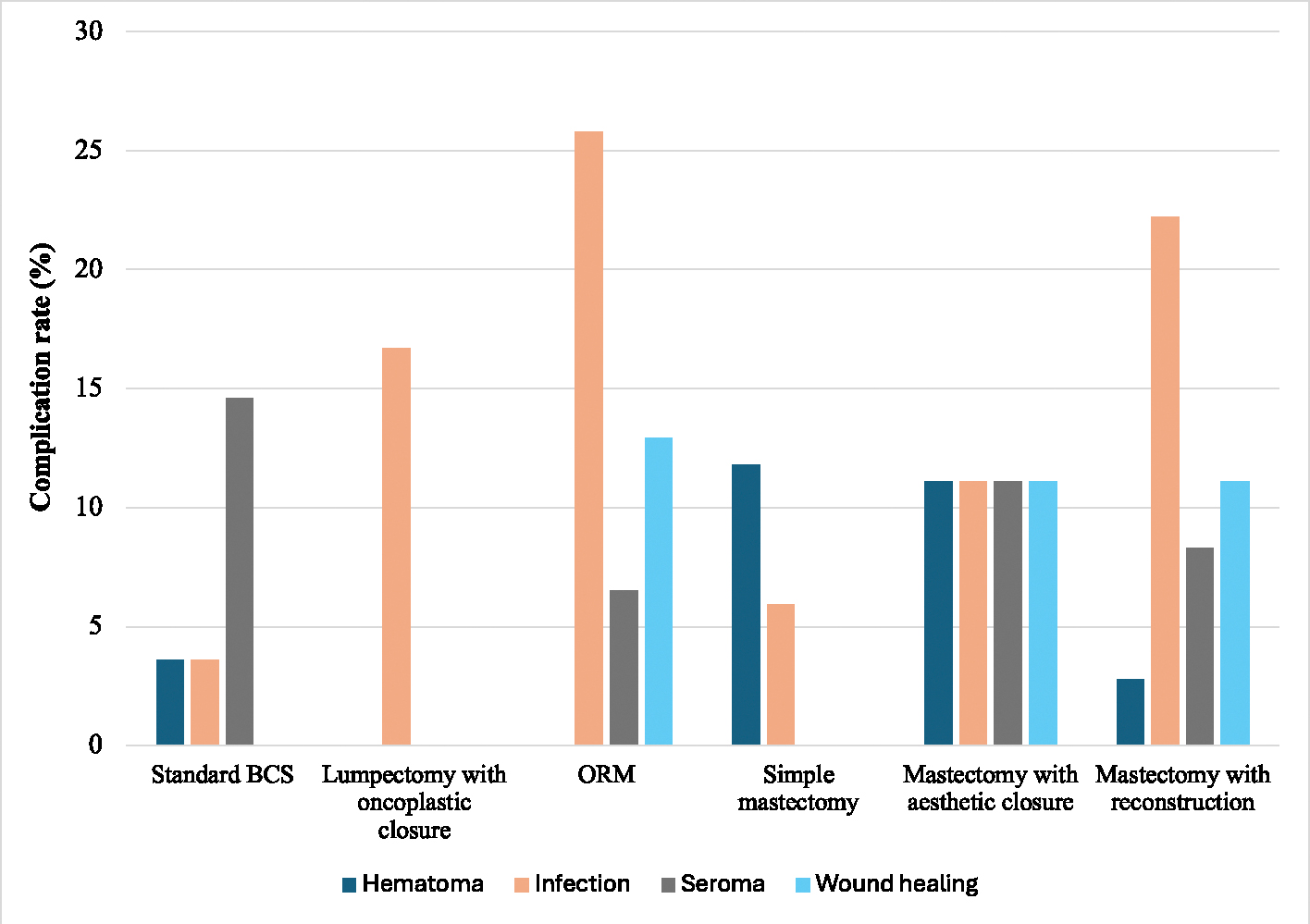
Incidence of each complication type by procedure.

Among BCS patients, while the complication rate was numerically higher in those undergoing ORM, there was no significant difference in overall complication rates between standard BCS, BCS with oncoplastic closure, and ORM (18.2 %, 16.7 %, and 35.5 %, p = 0.259) (Fig. 1). In the ORM group, surgical site infection was the most common complication, occurring in 25.8 % of cases, with wound healing complications being the second most common at 12.9 % (Fig. 2).

Among mastectomy patients, there was no statistically significant difference in overall complication rate between simple mastectomy, mastectomy with aesthetic flat closure, and mastectomy with reconstruction (11.8 %, 33.3 %, and 27.9 % respectively, p = 0.064) (Fig. 1). When further stratifying the cohort of patients with mastectomy with reconstruction, SSM had a higher overall complication rate compared to TSSM, simple mastectomy, and mastectomy with aesthetic closure (42.1 % vs 11.8 %, 11.8 %, and 33.3 %, p = 0.085). Of note, autologous reconstruction was more common in the SSM group than the TSSM group (42.1 % versus 5.9 %). Notably, one patient with autologous reconstruction experienced tissue flap necrosis following mastectomy with reconstruction, and one patient with implant-based reconstruction experienced skin flap necrosis following mastectomy with reconstruction. Both of these complications were categorized as “wound healing” complications. Furthermore, among SSM and TSSM patients, immediate placement of a tissue expander was associated with a numerically but not significantly lower overall complication rate compared to those who underwent immediate autologous reconstruction (22.2 % versus 44.4 %, p = 0.197).

Across all procedures, 15 patients required at least one unplanned reoperation due to surgical complications. Among these patients, the average number of reoperations was 1.75 (range 1–6 reoperations). Patients who underwent BCS as their initial oncologic procedure were significantly less likely to require subsequent reoperations compared to patients who underwent mastectomy (3.3 % versus 19.4 %, p = 0.001). In particular, patients who initially underwent mastectomy with reconstruction accounted for 66.7 % of all unplanned reoperations.

Patients who underwent standard BCS, lumpectomy with oncoplastic closure, or simple mastectomy had the lowest complication rates (18.2 %, 16.7 %, and 11.8 %, respectively), while patients undergoing ORM, mastectomy with aesthetic closure, and SSM had the highest complication rates (35.5 %, 33.3 %, and 42.1 % respectively). Overall complication rates between procedures did not significantly differ by T stage. There was a significantly higher complication rates in patients who underwent any type of oncoplastic surgery or immediate reconstruction compared to those who did not undergo oncoplastic or reconstructive surgery (30.5 % versus 16.7 %, p = 0.045).

In a multivariate logistic regression model adjusting for age, shave margins, tumor stage, type of breast surgery (mastectomy versus BCS), use of oncoplastic surgery, presence of diabetes mellitus, and BMI, increased BMI was associated with a higher likelihood of post-operative complications (odds ratio = 1.84, 95 % confidence interval [1.21–2.8], p = 0.004).

### 3.3. Positive margin rates by procedure type and T category

**Fig. 3.**
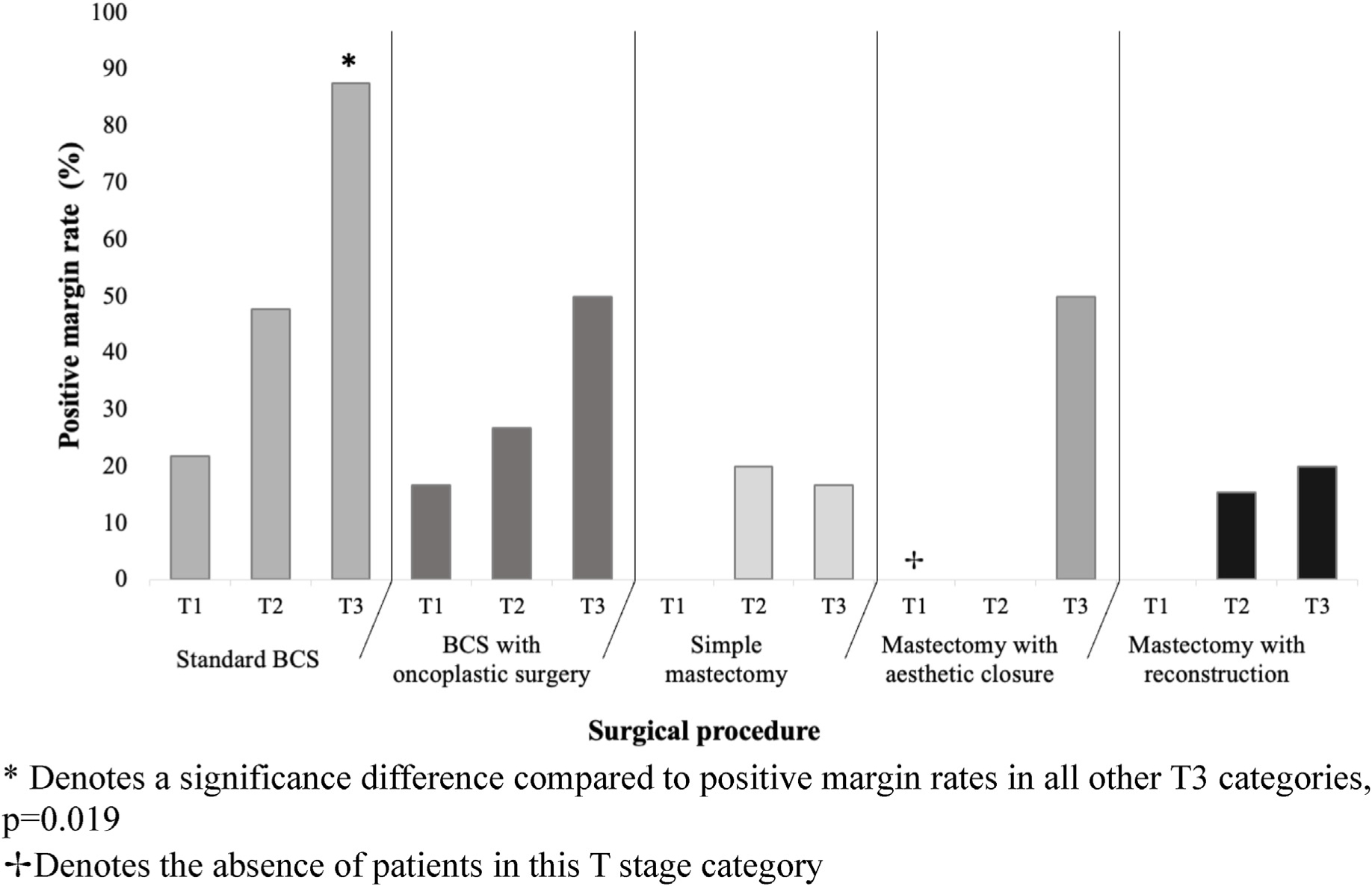
Positive margin rates across initial surgical procedures.

Margin data was available in 151 of 154 initial oncologic procedures. Of those, 43 cases (28.5 %) had positive margins. Across all procedure types, standard BCS had the highest rate of positive margins (42.6 %). The positive margin rate was significantly higher in patients who underwent standard BCS compared to those who underwent BCS with oncoplastic surgery, simple mastectomy, mastectomy with aesthetic flat closure, or mastectomy with immediate reconstruction (42.6 % versus 29.7 %, 13.3 %, 22.2 %, and 13.9 % respectively, p = 0.027). This difference was most notable among patients with T3 tumor category (Fig. 3). Overall, positive margin rates were significantly higher in patients who underwent any type of BCS compared to any type of mastectomy (37.4 % vs 15.0 %, p = 0.003). Shave margin use was not associated with lower positive margin rates among BCS patients.

On multivariate analysis of patients undergoing BCS, the use of oncoplastic surgery was significantly associated with reduced odds of positive margins when adjusting for patient age and tumor stage (odds ratio for oncoplastic surgery 0.3, 95 % confidence interval [0.1–0.9], p = 0.035).

### 3.4. Rates of subsequent oncologic procedures and associated complications

A total of 41 patients (26.6 %) underwent a second oncologic procedure. Among BCS patients, 34 (37.0 %) underwent a second oncologic procedure, and 5 patients required a third. Among mastectomy patients, only 7 patients (11.3 %) underwent a second oncologic operation, with none proceeding to a third.

The overall complication rate for all second oncologic operations was 14.6 %, with no significant difference in complication rates between patients who underwent BCS and those who had a mastectomy for their second oncologic procedure (7.7 % versus 26.7 %, p = 0.098).

Infection was the most common complication, occurring in 12.2 % of all second operations. Seroma and wound healing complications each accounted for 2.4 % of complications across all second operations, while hematoma did not occur in any cases. Among the 5 cases requiring a third operation, 1 case (20.0 %) was complicated by infection.

Of all BCS patients who underwent a second or third operation, 18 (52.9 %) ultimately underwent completion mastectomy for their final procedure, while 16 (47.1 %) underwent re-excision as their final procedure. For all mastectomy patients who underwent second operation, 6 (85.7 %) underwent re-excision, and 1 (14.3 %) underwent re-excision of nipple areolar complex with conversion to mastectomy and aesthetic flat closure due to residual disease.

### 3.5. Total number of operations

On average, patients underwent 1.7 total operations, encompassing all oncologic and additional reconstructive operations. The average number of oncologic operations was 1.3, and was significantly higher in the BCS group compared to the mastectomy group (1.4 versus 1.1, p < 0.001). The average number of oncoplastic/reconstructive operations was 0.4, and was lower in the BCS group compared to the mastectomy group (0.2 versus 0.7, p < 0.001). When evaluating the proportion of patients who had three or more total operations of any type, this proportion was significantly higher in those whose initial oncologic procedure was a mastectomy with reconstruction, in comparison to those who had standard BCS, lumpectomy with oncoplastic closure/ORM, simple mastectomy, or mastectomy with aesthetic flat closure (36.1 % versus 18.2 %, 8.1 %, 0 %, and 0 %, respectively, p = 0.002).

## 4. Discussion

These results demonstrate significant challenges with surgical management of ILC for those with BMI≥30 kg/m^2^, a group representing a high proportion of the population. Standard BCS was associated with lower risk of early surgical complications compared to BCS with oncoplastic approaches, but higher rates of positive margins. For those undergoing mastectomy, surgical complications were more common in patients undergoing mastectomy with reconstruction, particularly in the SSM group, where the higher rate of autologous reconstruction most likely contributed to the increased complication rates.

Patients with obesity who are diagnosed with breast cancer face undergoing oncologic surgery without time for pre-operative weight optimization. Because of increased risks of surgical complications in the setting of BMI≥30 kg/m^2^, surgical choices may be more limited. Indeed, the literature is replete with studies showing the association between elevated BMI and surgical complications, most notably after reconstructive procedures.^7,18–27^

Our rate of complications in this population of patients with stage I-III breast cancer and BMI≥30 kg/m^2^ is similar to those reported in the literature. In the setting of BCS, we found that 23.9 % had complications. Although data are limited, one study reported a 40 % complication rate among 199 patients with a BMI over 30 kg/m^2^ who underwent oncoplastic breast reduction. Similarly, another study of 182 patients that received bilateral reduction mammoplasty reported an overall complication rate of 51 %.^28,29^

For those undergoing mastectomy, our overall complication rate of 22.6 % is also consistent with prior studies. Complication rates for those undergoing mastectomy in the setting of BMI ≥30 kg/m^2^ range from as low as 7.5 % for simple mastectomy, to 26.5 % in those undergoing reconstruction.

Interestingly, the small number of patients in our series who underwent aesthetic flat closure had a higher complication rate than we expected, as well as a higher positive margin rate. This is a procedure in which there is growing interest, which involves de-epithelialization and rotation of skin flaps to create an aesthetic chest wall contour. In our population, 33 % of patients experienced a post-operative complication; in a previously published series of 10 patients with morbid obesity who underwent this procedure 40 % had wound healing complications.^30^ The high positive margin rate seen in patients with T3 ILC raises the question of the oncologic safety of this procedure in this population, and whether preservation of skin for chest wall contouring inadvertently resulted in retained breast tissue; with only 9 patients we cannot draw conclusions, but our results suggest the need for further study into aesthetic flat closure for patients with large ILC tumors.

Although we found that oncoplastic and reconstructive procedures were associated with higher rates of surgical complications compared to standard BCS or simple mastectomy, these findings must be balanced with the risk of positive margins. For patients undergoing BCS, there was a strong association between oncoplastic surgery and lower risk of positive margins. Among those undergoing mastectomy, however, simple mastectomy (without aesthetic closure or reconstruction) was associated with the lowest risk of positive margins. There are limited data in the literature to compare these findings; our prior analysis of this ILC database (including all BMI groups) found no difference in positive margin rates by type of mastectomy.^31^ However, in that analysis we did not identify patients who had aesthetic flat closure specifically, but rather grouped them with mastectomy without reconstruction. Whether the preservation of skin and soft tissue in aesthetic flat closure leads to increased risk of positive margins for those with ILC deserves further attention and study.

In our study, a high proportion of patients had shave margins taken during both BCS and mastectomy procedures. While we previously showed that shave margin use was associated with reduced positive margins in patients with ILC, we did not observe this relationship in this analysis.^32^ The high rates of positive margins despite the use of shave margins reflect the challenges of surgical resection for diffusely growing tumors like ILC where imaging often underestimates tumor size. Studies have reported positive margin rates as high as 60 % following BCS for ILC, with one study by Wanis et al. demonstrating re-excision rates around 35.1 % for positive or close margins.^9,13,14,33,34^ In mastectomy cases, the literature suggests positive margin rates ranging from 3 to 27 % depending on the definition of margin width utilized.^35–40^ In our study, patients who underwent BCS had a positive margin rate of 37.4 %, compared to 15.0 % in those who underwent mastectomy. Notably, 19.6 % of the BCS population had T3 tumors, a tumor size which is traditionally considered a contraindication to breast conservation. This may have contributed to higher positive margin rates.

When evaluating surgical complications and positive margin rates in isolation, our study would suggest that simple mastectomy may be the optimal surgical procedure for patients with ILC and BMI ≥30 kg/m^2^. However, several other factors influence surgical decision making, including patient preference.

Advantages of BCS include avoidance of mastectomy, and the impact of post-mastectomy radiotherapy on a reconstructed breast, which is associated with risk of complications particularly for those undergoing implant-based reconstruction. Additionally, we saw that unplanned returns to the operating room were less common in those undergoing BCS.

While these surgical complications are unlikely to be related to the histologic subtype of breast cancer, the risk of positive margins is known to be elevated in ILC specifically. As such, for those with this tumor type, surgical decision making must include considerations of patient preferences for breast conservation, risk of positive margins associated with each surgical approach, as well as risk of surgical complications. Despite the risk of surgical complications, with post-operative infection requiring antibiotics being the most common, we feel that BCS with oncoplastic approaches still provides a reasonable choice for patients with ILC and BMI ≥30 kg/m^2^ given its association with lower positive margin rates and avoidance of mastectomy.

Our study showed an increased risk for positive margins across all procedure types for patients with higher T stage, particularly pronounced for those undergoing BCS. However, we observed a significant reduction in the risk of positive margins among BCS patients when ORM or oncoplastic approach was used. Previous research from our institution has also demonstrated that ORM yields comparable positive margin rates and overall outcomes compared to mastectomy.^32^ This suggests that employing an oncoplastic approach with BCS may be a preferable option for patients with T1-T2 ILC, especially those with elevated BMI. Conversely, we found that T3 stage disease exhibited the highest positive margin rates with BCS procedures, ranging from 50 to 87.5 % for lumpectomy and lumpectomy with oncoplastic approaches. These findings align with existing literature, which have consistently demonstrated the high likelihood of positive margins when attempting BCS in patients with T3 disease.^41^ Thus, for T3 cases, some may consider mastectomy a more suitable choice but the high risk of positive margins still persists, reflecting the growth pattern of these tumors. Accordingly, well-informed patients may still opt for BCS with oncoplastic surgery over mastectomy even in setting of T3 tumor.

While we were unable to evaluate the impact of radiation on outcomes, the growing indications for post-mastectomy radiation should also be considered for those with larger tumors, who may experience higher rates of complications if radiotherapy is received after mastectomy with reconstruction versus after BCS with oncoplastic surgery.

### 4.1. Limitations and future directions

Given the retrospective nature of this study we recognize the inherent potential for selection bias and incomplete data capture. The single institution design may also limit the generalizability of our findings to broader populations. Furthermore, the limited sample size in certain subgroups may have reduced our ability to detect statistical differences. Another potential limitation of this study is the extended data collection period, which may capture evolving surgical practices. At our institution, oncoplastic techniques were primarily adopted after 2000, with an increase over time in its use. Although the large time period introduces heterogeneity, these operations were performed by a small group of plastic surgeons, with no major changes to the institutional approach during the study period. Lastly, the absence of a comparison group of patients with lower BMI in our study design prevents direct comparisons, limiting our ability to explore distinctions between individuals with elevated and lower BMI. However, given the lack of research on surgical complications specifically in ILC patients with elevated BMI, we believe that our results represent an important piece of evidence to assist in optimizing outcomes for this patient population.

## 5. Conclusion

In this cohort of ILC patient with elevated BMI, we found that 23.4 % experienced a surgical complication after their first oncologic surgery, with a higher risk in those who underwent oncoplastic surgery or immediate reconstruction. Although BCS procedures had the highest positive margin rates, these rates were significantly reduced when ORM was utilized as the BCS procedure. Simple mastectomy had the lowest overall complication rate and positive margin rate, yet this procedure may not align with patient goals for breast conservation or reconstruction. Finally, patients who underwent mastectomy with immediate reconstruction as their first oncologic surgery had significantly more subsequent operations and unplanned reoperations on average compared to other initial procedures. These findings should be used for shared decision making to help patients with ILC and elevated BMI select the surgical procedure that most aligns with their goals and preferences.

## Figures and Tables

**Table 1:** Demographic and clinicopathologic features in patients with initial BCS compared to mastectomy.

	Total	BCS	Mastectomy	p-value
				
	N = 154	N = 92	N = 62	

Age at diagnosis	62.9 (11.6)	63.5 (10.9)	62.1 (12.7)	0.44
BMI (kg/m^2^)	34.9 (4.7)	35.4 (4.7)	34.2 (4.5)	0.12
ILC receptor subtype				0.072
ER + PR + HER−	129 (86.0 %)	79 (87.8 %)	50 (83.3 %)	
ER + PR−HER−	17 (11.3 %)	7 (7.8 %)	10 (16.7 %)	
HER2+	4 (2.7 %)	4 (4.4 %)	0 (0.0 %)	
ILC grade				0.60
1	36 (23.8 %)	24 (26.7 %)	12 (19.7 %)	
2	104 (68.9 %)	60 (66.7 %)	44 (72.1 %)	
3	11 (7.3 %)	6 (6.7 %)	5 (8.2 %)	
ILC tumor diameter (cm)	3.8 (3.0)	3.0 (2.2)	5.1 (3.5)	<0.001
Overall stage				0.034
1	37 (24.2 %)	28 (30.8 %)	9 (14.5 %)	
2	85 (55.6 %)	49 (53.8 %)	26 (58.1 %)	
3	31 (20.3 %)	14 (15.4 %)	17 (27.4 %)	
T stage				0.003
1	48 (31.4 %)	36 (39.1 %)	12 (19.7 %)	
2	61 (39.9 %)	38 (41.3 %)	23 (37.7 %)	
3	44 (28.8 %)	18 (19.6 %)	26 (42.6 %)	
Shave margins used	85 (64.9 %)	65 (76.5 %)	20 (43.5 %)	<0.001
Lymph node status				0.14
Node negative	97 (63.4 %)	62 (68.1 %)	35 (56.5 %)	
Node positive	56 (36.6 %)	29 (31.9 %)	27 (43.5 %)	
Surgery 1 positive margins	42 (27.5 %)	34 (37.4 %)	8 (12.9 %)	<0.001

**Procedure types**				

Surgery 1				<0.001
Lumpectomy	55 (35.7 %)	55 (59.8 %)	0 (0.0 %)	
Lumpectomy with oncoplastic closure	6 (3.9 %)	6 (6.5 %)	0 (0.0 %)	
ORM	31 (20.1 %)	31 (33.7 %)	0 (0.0 %)	
Simple mastectomy	17 (11.0 %)	0 (0.0 %)	17 (27.4 %)	
Mastectomy with aesthetic closure	9 (5.8 %)	0 (0.0 %)	9 (14.5 %)	
SSM	19 (12.3 %)	0 (0.0 %)	19 (30.1 %)	
TSSM	17 (11.0 %)	0 (0.0 %)	17 (27.4 %)	
Surgery 2				0.079
Re-excision	21 (51 %)	16 (47 %)	5 (71 %)	
Lumpectomy	3 (7 %)	2 (6 %)	1 (14 %)	
Lumpectomy with oncoplastic closure	1 (2 %)	1 (3 %)	0 (0 %)	
ORM	1 (2 %)	1 (3 %)	0 (0 %)	
Simple mastectomy	6 (15 %)	6 (18 %)	0 (0 %)	
Mastectomy with aesthetic closure	1 (2 %)	0 (0 %)	1 (14 %)	
SSM	5 (12 %)	5 (15 %)	0 (0 %)	
TSSM	3 (7 %)	3 (9 %)	0 (0 %)	
Surgery 3				
Re-excision	1 (20 %)	1 (20 %)	0 (0 %)	
Simple mastectomy	1 (20 %)	1 (20 %)	0 (0 %)	
SSM	2 (40 %)	2 (40 %)	0 (0 %)	
TSSM	1 (20 %)	1 (20 %)	0 (0 %)	

**Surgery 1 complication types**				

Any complication	37 (24.0 %)	22 (23.9 %)	15 (24.2 %)	0.97
Hematoma	6 (3.9 %)	2 (2.2 %)	4 (6.5 %)	0.18
Infection	21 (13.6 %)	11 (12.0 %)	10 (16.1 %)	0.46
Seroma	14 (9.1 %)	10 (10.9 %)	4 (6.5 %)	0.35
Wound healing	9 (5.0 %)	4 (4.3 %)	5 (8.1 %)	0.33
Reoperation	15 (9.7 %)	3 (3.3 %)	12 (19.4 %)	<0.001

Data are presented as mean (SD) for continuous measures, and n (row %) for categorical measures and n (column %) for binary measures. Not all patients had complete demographic information, leading to varying sample sizes for certain variables.

## References

[1] Czajka ML, Pfeifer C. Breast cancer surgery. In: StatPearls. StatPearls Publishing; 2024. http://www.ncbi.nlm.nih.gov/books/NBK553076/. Accessed March 13, 2024. 31971717

[2] Hammer C, Fanning A, Crowe J. Overview of breast cancer staging and surgical treatment options. Cleve Clin J Med. 2008;75(Suppl 1):S10–S16. 10.3949/ccjm.75.suppl_1.s10. 18457192

[3] Mohamedahmed AYY, Zaman S, Zafar S, Comparison of surgical and oncological outcomes between oncoplastic breast-conserving surgery versus conventional breast-conserving surgery for treatment of breast cancer: a systematic review and meta-analysis of 31 studies. Surg Oncol. 2022;42:101779. 10.1016/j.suronc.2022.101779. 35567982

[4] de Boniface J, Szulkin R, Johansson ALV. Medical and surgical postoperative complications after breast conservation versus mastectomy in older women with breast cancer: Swedish population-based register study of 34 139 women. Br J Surg. 2023;110(3):344–352. 10.1093/bjs/znac411. 36511352 PMC10364521

[5] Bryan S, Afful J, Carroll M, NHSR 158. National Health and Nutrition Examination Survey 2017–March 2020 Pre-pandemic Data Files. National Center for Health Statistics (U.S.); 2021. 10.15620/cdc:106273. PMC11513744 39380201

[6] Lee K, Kruper L, Dieli-Conwright CM, Mortimer JE. The impact of obesity on breast cancer diagnosis and treatment. Curr Oncol Rep. 2019;21(5):41. 10.1007/s11912-019-0787-1. 30919143 PMC6437123

[7] Srinivasa DR, Clemens MW, Qi J, Obesity and breast reconstruction: complications and patient-reported outcomes in a multicenter, prospective study. Plast Reconstr Surg. 2020;145(3):481e–490e. 10.1097/PRS.0000000000006543. 32097295

[8] Cevallos P, Berry C, Lipman KJ, Breast reconstruction after mastectomy in patients with obesity: a narrative review. Ann Transl Med. 2023;11(12):413. 10.21037/atm-23-1599. 38213816 PMC10777214

[9] Moore MM, Borossa G, Imbrie JZ, Association of infiltrating lobular carcinoma with positive surgical margins after breast-conservation therapy. Ann Surg. 2000;231(6):877–882. 10816631 10.1097/00000658-200006000-00012 PMC1421077

[10] Luveta J, Parks RM, Heery DM, Cheung KL, Johnston SJ. Invasive lobular breast cancer as a distinct disease: implications for therapeutic strategy. Oncol Ther. 2019;8(1):1–11. 10.1007/s40487-019-00105-0. 32700069 PMC7359988

[11] Sagara Y, Barry WT, Mallory MA, Surgical options and locoregional recurrence in patients diagnosed with invasive lobular carcinoma of the breast. Ann Surg Oncol. 2015;22(13):4280–4286. 10.1245/s10434-015-4570-8. 25893416 PMC4801503

[12] Talsma AK, Reedijk AMJ, Damhuis RaM, Westenend PJ, Vles WJ. Re-resection rates after breast-conserving surgery as a performance indicator: introduction of a case-mix model to allow comparison between Dutch hospitals. Eur J Surg Oncol. 2011;37(4):357–363. 10.1016/j.ejso.2011.01.008. 21292434

[13] Wanis ML, Wong JA, Rodriguez S, Rate of re-excision after breast-conserving surgery for invasive lobular carcinoma. Am Surg. 2013;79(10):1119–1122. 24160812

[14] Mai KT, Yazdi HM, Isotalo PA. Resection margin status in lumpectomy specimens of infiltrating lobular carcinoma. Breast Cancer Res Treat. 2000;60(1):29–33. 10.1023/a:1006359308505. 10845806

[15] Piper ML, Wong J, Fahrner-Scott K, Success rates of re-excision after positive margins for invasive lobular carcinoma of the breast. NPJ Breast Cancer. 2019;5:29. 10.1038/s41523-019-0125-7. 31508489 PMC6731236

[16] Sakr RA, Poulet B, Kaufman GJ, Nos C, Clough KB. Clear margins for invasive lobular carcinoma: a surgical challenge. Eur J Surg Oncol. 2011;37(4):350–356. 10.1016/j.ejso.2011.01.010. 21277728

[17] Obesity WPRO. https://www.who.int/westernpacific/health-topics/obesity. Accessed April 14, 2024.

[18] Al-Mulhim AS, Al-Hussaini HA, Al-Jalal BA, Al-Moagal RO, Al-Najjar SA. Obesity disease and surgery. Int J Chronic Dis. 2014;2014:652341. 10.1155/2014/652341. 26464861 PMC4590927

[19] de Blacam C, Ogunleye AA, Momoh AO, High body mass index and smoking predict morbidity in breast cancer surgery: a multivariate analysis of 26,988 patients from the national surgical quality improvement program database. Ann Surg. 2012;255(3):551–555. 10.1097/SLA.0b013e318246c294. 22330036

[20] Wang M, Huang J, Chagpar AB. Do obese breast cancer patients have more complications and a longer length of stay after mastectomy than nonobese patients? Am Surg. 2021;87(7):1099–1106. 10.1177/0003134820973352. 33316161

[21] Rezaei SJ, Boskey ER, Ganor O. Body mass index and benign breast surgeries: a survey of plastic surgeons’ knowledge and attitudes. JPRAS Open. 2023;36:46–54. 10.1016/j.jpra.2023.02.001. 37102187 PMC10123250

[22] Chen CL, Shore AD, Johns R, Clark JM, Manahan M, Makary MA. The impact of obesity on breast surgery complications. Plast Reconstr Surg. 2011;128(5):395e–402e. 10.1097/PRS.0b013e3182284c05. 21666541

[23] Valente DS, Zanella RK, Mulazzani CM, Valente SS. Risk factors for explantation of breast implants: a cross-sectional study. Aesthetic Surg J. 2021;41(8):923–928. 10.1093/asj/sjaa352. 33649754

[24] Garland M, Hsu FC, Clark C, Chiba A, Howard-McNatt M. The impact of obesity on outcomes for patients undergoing mastectomy using the ACS-NSQIP data set. Breast Cancer Res Treat. 2018;168(3):723–726. 10.1007/s10549-017-4651-4. 29327298

[25] Cuccolo NG, Kang CO, Boskey ER, Mastectomy in transgender and cisgender patients: a comparative analysis of epidemiology and postoperative outcomes. Plast Reconstr Surg Glob Open. 2019;7(6):e2316. 10.1097/GOX.0000000000002316. 31624695 PMC6635198

[26] Myung Y, Heo CY. Relationship between obesity and surgical complications after reduction mammaplasty: a systematic literature review and meta-analysis. Aesthetic Surg J. 2017;37(3):308–315. 10.1093/asj/sjw189. 28207040

[27] Gupta V, Yeslev M, Winocour J, Aesthetic breast surgery and concomitant procedures: incidence and risk factors for major complications in 73,608 cases. Aesthetic Surg J. 2017;37(5):515–527. 10.1093/asj/sjw238. 28333172

[28] Fortenbery GW, Todd L, Nazir N, Dalla S, Collins M. Oncoplastic breast reconstruction in morbidly obese patients: an acceptable practice. Plast Reconstr Surg Glob Open. 2024;12(2):e5601. 10.1097/GOX.0000000000005601. 38348460 PMC10861004

[29] Hinson C, Alford H, Huett W, Obesity and complications in mammoplasty: a retrospective review in an obese patient population. Plastic and Reconstructive Surgery Global Open. 2022;10(12). 10.1097/GOX.0000000000004697. PMC9742115 36518689

[30] Schwartz JCD. Single-incision approach to aesthetic flat closure after bilateral mastectomy in morbidly obese patients. JPRAS Open. 2023;39:18–22. 10.1016/j.jpra.2023.10.014. 38107034 PMC10724483

[31] Hewitt KC, Miller P, Piper M, Positive margins after mastectomy in patients with invasive lobular carcinoma of the breast: incidence and management strategies. Am J Surg. 2022;223(4):699–704. 10.1016/j.amjsurg.2021.05.021. 34148670

[32] Mukhtar RA, Wong J, Piper M, Breast conservation and negative margins in invasive lobular carcinoma: the impact of oncoplastic surgery and shave margins in 358 patients. Ann Surg Oncol. 2018;25(11):3165–3170. 10.1245/s10434-018-6682-4. 30054826

[33] Arps DP, Jorns JM, Zhao L, Bensenhaver J, Kleer CG, Pang JC. Re-excision rates of invasive ductal carcinoma with lobular features compared with invasive ductal carcinomas and invasive lobular carcinomas of the breast. Ann Surg Oncol. 2014;21(13):4152–4158. 10.1245/s10434-014-3871-7. 24980090

[34] Wilke LG, Czechura T, Wang C, Repeat surgery after breast conservation for the treatment of stage 0 to II breast carcinoma: a report from the National Cancer Data Base, 2004–2010. JAMA Surg. 2014;149(12):1296–1305. 10.1001/jamasurg.2014.926. 25390819

[35] Sledge GW, Chagpar A, Perou C. Collective wisdom: lobular carcinoma of the breast. American Society of Clinical Oncology Educational Book. 2016;(36):18–21. 10.1200/EDBK_100002. 27249682

[36] Donovan CA, Harit AP, Chung A, Bao J, Giuliano AE, Amersi F. Oncological and surgical outcomes after nipple-sparing mastectomy: do incisions matter? Ann Surg Oncol. 2016;23(10):3226–3231. 10.1245/s10434-016-5323-z. 27352202

[37] Rowell NP. Are mastectomy resection margins of clinical relevance? A systematic review. Breast. 2010;19(1):14–22. 10.1016/j.breast.2009.10.007. 19932025

[38] Yu J, Al Mushawah F, Taylor ME, Compromised margins following mastectomy for stage I-III invasive breast cancer. J Surg Res. 2012;177(1):102–108. 10.1016/j.jss.2012.03.046. 22520579 PMC3924779

[39] Kataoka A, Sawaki M, Okumura S, Prediction of pathological margin status using preoperative contrast-enhanced MRI in patients with early breast cancer who underwent skin-sparing mastectomy. Breast J. 2019;25(2):202–206. 10.1111/tbj.13194. 30697867

[40] Sheikh F, Rebecca A, Pockaj B, Inadequate margins of excision when undergoing mastectomy for breast cancer: which patients are at risk? Ann Surg Oncol. 2011;18(4):952–956. 10.1245/s10434-010-1406-4. 21080087

[41] Wj H, As E, Js R, C P, Dh B. Rates of margin positive resection with breast conservation for invasive breast cancer using the NCDB. Breast. 2021;60:86–89. 10.1016/j.breast.2021.08.012. 34520952 PMC8441089

